# Vitiligo and melanocytic nevi: New findings in Coffin-Siris syndrome associated with *ARID1* germline mutation

**DOI:** 10.1016/j.jdcr.2018.08.024

**Published:** 2018-12-05

**Authors:** Catherine N. Tchanque-Fossuo, Sara E. Dahle, Maija Kiuru, R. Rivkah Isseroff

**Affiliations:** a Department of Dermatology, University of California, Davis, Sacramento, California; b Dermatology Service, VA Northern California, Sacramento VA Medical Center, Mather, California; c Podiatry Section, Department of Surgery, VA Northern California, Sacramento VA Medical Center, Mather, California; d Department of Pathology and Laboratory Medicine, University of California, Davis, Sacramento, California

**Keywords:** *ARID1B*, Coffin-Siris syndrome, melanoma, nevi, vitiligo, *ARID1A*, AT-rich interactive domain 1A gene, *ARID1B*, AT-rich interactive domain 1B gene, CDK, cyclin-dependent kinase, CSS, Coffin-Siris syndrome, MITF, microphthalmia transcription factor, *SMARCA4*, SWI/SNF–related, matrix-associated, actin-dependent regulator of chromatin subfamily A member 4, *SMARB1*, SWI/SNF–related, matrix-associated, actin-dependent regulator of chromatin subfamily B member 1, *SMARCE1*, SWI/SNF–related, matrix-associated, actin-dependent regulator of chromatin subfamily E member 1, SWI/SNF, switch/sucrose nonfermenting

## Introduction

Coffin-Siris syndrome (CSS; Mendelian Inheritance in Man database no. 135900) is a rare autosomal dominant syndrome characterized by impaired cognition, developmental delay, hypoplasia of the fifth digit toe nail, thick eyebrows, broad nasal bridge, wide mouth, hirsutism, microcephaly, short stature, and organ dysfunction.^1^, ^2^, ^3^ There are only 80 cases reported since it was first described in 1970.^1^ CSS is associated with haploinsufficiency, typically due to a de novo germline mutation in 1 of 6 genes: AT-rich interactive domain 1A (*ARID1A*) or 1B (*ARID1B*); switch/sucrose nonfermenting (SWI/SNF)–related, matrix-associated, actin-dependent regulator of chromatin subfamily A member 4 (*SMARCA4*), subfamily B member 1 (*SMARCB1*), or subfamily E member 1 (*SMARCE1*); or sex-determining region–related HMG-box 11.^1^, ^2^, ^3^

## Case report

A 15-year-old boy presented with multiple asymptomatic cutaneous white patches, noticed by his mother 4 years before presentation. The mother's pregnancy and patient's birth history were unremarkable. The patient had difficulty feeding since birth, laryngomalacia requiring gastric tube placement, and recurrent hospitalizations for aspiration pneumonia.

His clinical features of bushy arched eyebrows, thick everted lips, broad nasal bridge, facial nevi, and developmental delay prompted metabolic and genetic testing. At 11 years old, a diagnosis of CSS was established via whole-exome sequencing, revealing a de novo mutation (c.4202G>T, p.E1402X) in the *ARID1B* gene, a premature stop codon mutation leading to a truncated protein. This mutation was not found in either parent or his 2 unaffected older sisters.

**Fig 1:**
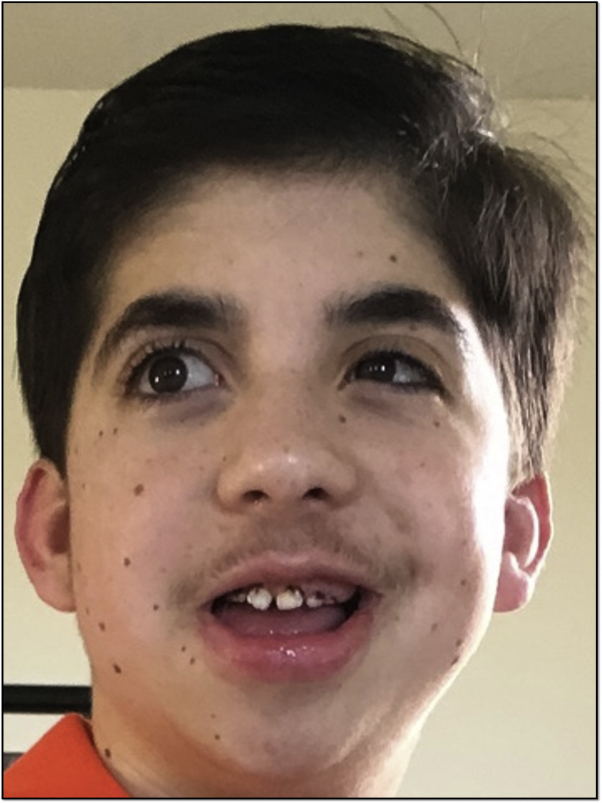
Clinical photographs of patient's face. Note the distinctive facial features with bushy arched eyebrows, long eyelashes, thick everted upper lip, broad nasal bridge, facial nevi, and hypertrichosis on upper cutaneous lip.

**Fig 2:**
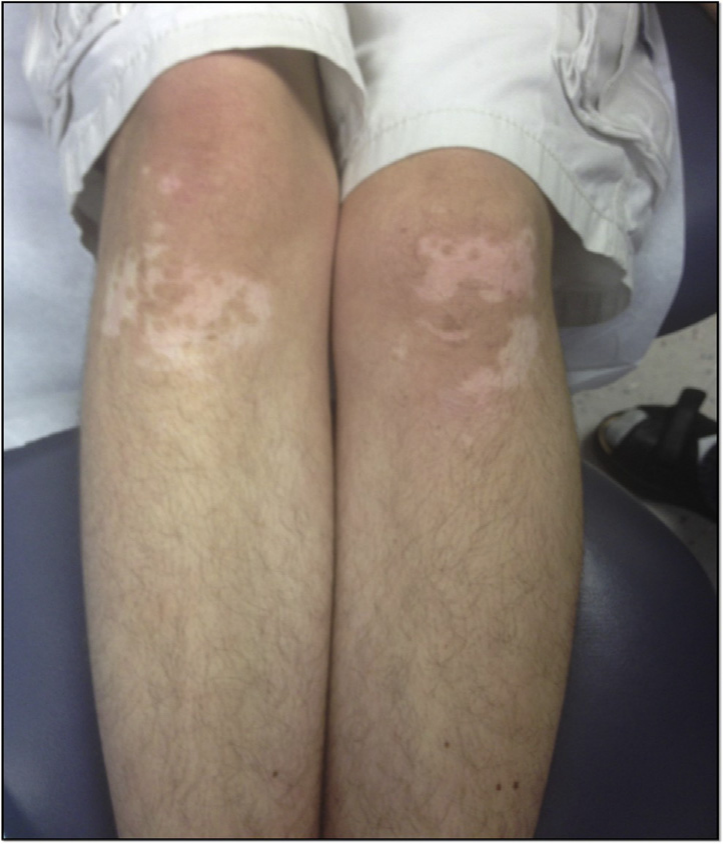
Clinical photograph of patient's knees, with numerous depigmented demarcated patches in a semisymmetrical distribution.

**Fig 3:**
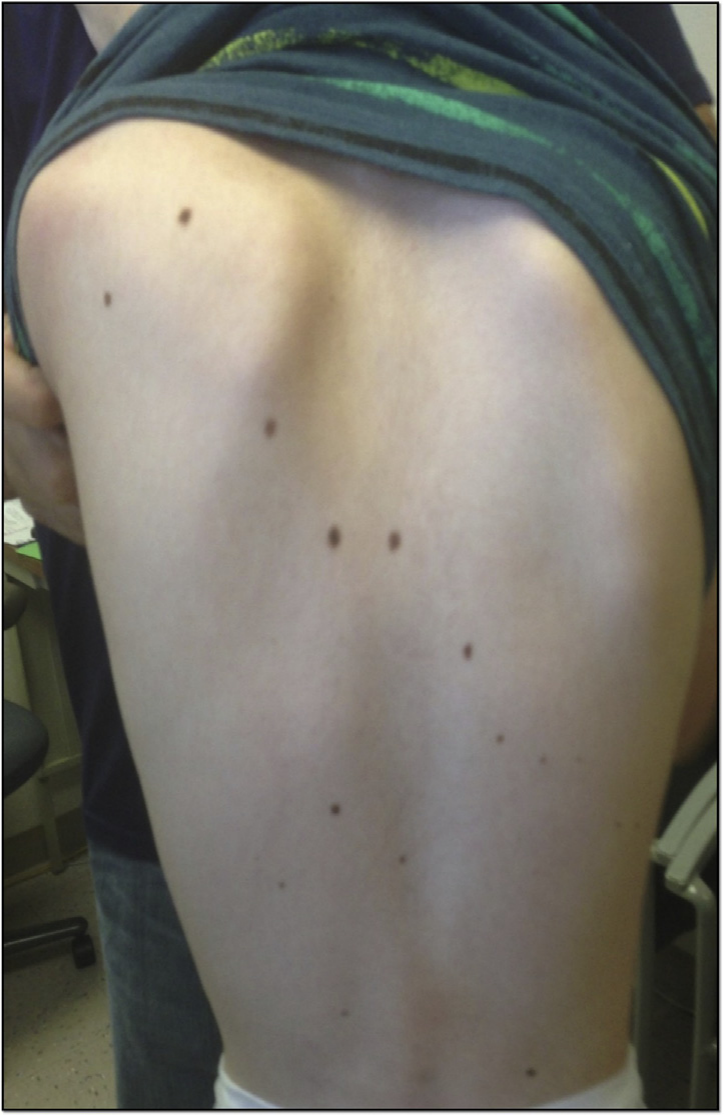
Clinical photograph of patient's trunk, with numerous scattered melanocytic nevi.

Clinical examination showed distinctive coarse facial features (Fig 1), fifth finger hypoplasia, first metatarsal hypermobility, and hypertrichosis characteristic of CSS. There were depigmented macules and patches on the elbows and knees (Fig 2) and malleoli, clinically characteristic of vitiligo. He had over 60 brown macules consistent with melanocytic nevi (under 5 mm) on the face (Fig 1), neck, abdomen, trunk (Fig 3), extremities, palms, and soles (up to 1.0 cm). Halo nevi were not present. The parents noted the onset of nevi around age 3 years, and neither had multiple nevi.

## Discussion

Most of the 6 identified genes causative of CSS (*ARID1A*, *ARID1B*, *SMARCA4*, *SMARCB1*, *SMARCE1*) encode for the Brahma-associated factor complex.^1^, ^2^, ^3^ These genes are subunits of the evolutionarily conserved ATP-dependent chromatin-remodeling complex SWI/SNF.^1^, ^2^, ^3^ The SWI/SNF pathway is essential for cellular transcription and differentiation, DNA repair, and tumor suppression.^3^ SWI/SNF functions as an epigenetic modifier by altering chromatin structure and, thereby, gene expression. SWI/SNF is the most commonly mutated chromatin-regulatory complex in human cancer, with a frequency of 20%, close to that of *p53* mutations.^3^ CSS patients have been found to harbor various tumors with these mutations, including hepatoblastoma (*ARID1* pathogenic variant),^3^ papillary thyroid cancer (deletion in *ARID1B*),^3^, ^4^ medulloblastoma (*SMARCA4*), meningiomas, and schwannomatosis (*SMARCB1* pathogenic variant).^3^, ^5^

*ARID1B* mutations are associated with a range of phenotypes in CSS patients.^4^ A series of 63 cases showed that the most common manifestations included intellectual disability, hypotonia, feeding problems, hypertrichosis, sparse scalp hair, thick eyebrows, long eyelashes, thick alae nasi, thick lower vermillion, malformed ears, lax joints, short fifth finger, and underdeveloped nails.^4^ In this large series of CSS individuals with *ARID1B* mutations, no vitiligo or prominent nevus phenotype were reported. Given the aforementioned clinical features, vitiligo might be a coincidental finding in our patient. However, it is plausible that pigmentary abnormalities or pigmented lesions could have been overlooked in this cohort. Indeed, a literature search using “vitiligo” and “Coffin Siris syndrome” key words, retrieved no reports on vitiligo in CSS patients.

**Fig 4:**
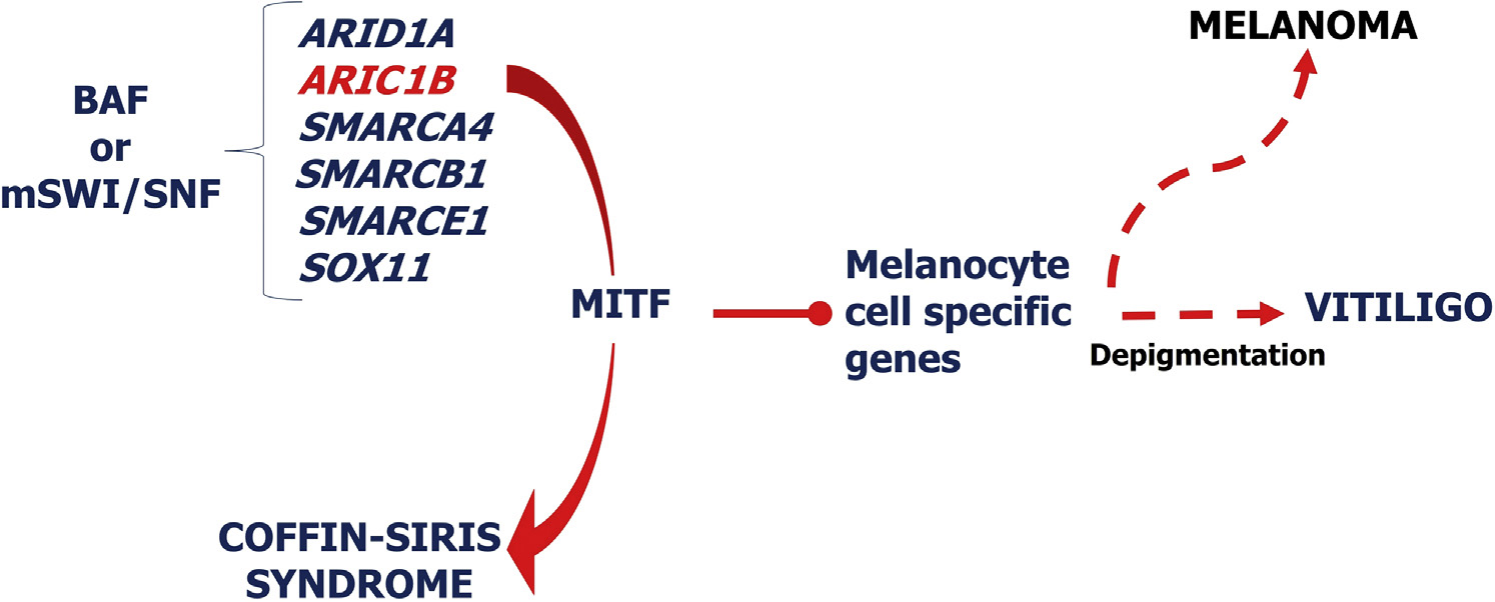
Schematic of role of switch/sucrose nonfermenting in the pathogenesis of Coffin Siris syndrome, vitiligo, and potential role in melanocytic nevi transformation into melanoma. *ARID1A*, AT-rich interactive domain 1A; *ARID1B*, AT-rich interactive domain 1B; *BAF*, Brahma-associated factor; *MITF*, microphthalmia transcription factor; *SMARCA4*, SWI/SNF–related, matrix-associated, actin-dependent regulator of chromatin subfamily A member 4; *SMARB1*, SWI/SNF–related, matrix-associated, actin-dependent regulator of chromatin subfamily B member 1; *SMARCE1*, SWI/SNF–related, matrix-associated, actin-dependent regulator of chromatin subfamily E member 1; *SOX11*, sex-determining region–related HMG-box 11; *SWI/SNF*, switch/sucrose nonfermenting.

We suggest, however, that there are genes and pathways shared between CSS, vitiligo, and melanocytic nevi. Specifically, since subunits of the SWI/SNF complex associate with the protein product of the microphthalmia transcription factor (*MITF*) gene,^6^ described as a master gene for survival of melanocytes and key transcription factor regulating melanin synthesis, mutations in the SWIF genes present in CSS could affect the transcription of melanin. MITF has various isoforms^7^ and mutations associated with a range of phenotypes in pigmented cells.^7^ Melanocytic isoform M mutations lead to Waardenburg syndrome type 2A, an auditory pigmented syndrome with hearing loss, patchy depigmentation of the skin, and irides.^7^ In contrast, a dominant-negative mutation causes Tietze syndrome, characterized by congenital deafness and generalized albinoid-like hypopigmentation of the skin, eyes, and hair.^7^ However, there are no data that currently point to a distinctive mutation on the *MITF* that would critically cause its dysfunction. Herein, we suggest that the mutations in *ARID1A*, *ARID1B*, *SMARCA4*, *SMARCB1*, and *SMARCE1* (causative of CSS) might create some interference that would lead to disturbances in the interaction between the SWI/SNF activity and MITF and alterations in MITF-regulated processes, such as the transcription of the 3 main enzymes involved in melanogenesis (Fig 4).

Melanomas show mutations in components of the SWI/SNF complex, such as *ARID1A*, *ARID1B*, *ARID2*, and *SMARCA4*.^8^, ^9^ Mutations in components of the SWI/SNF complex have been shown to be associated with tumor progression in melanomas.^10^ We postulate that the *ARID1B* germline mutation in our patient drives the development of melanocytic nevi, similar to *SMARCB1* germline mutations driving multiple schwannomas in a patient with CSS phenotype.^5^

A hereditary predisposition to melanoma is most commonly caused by a heterozygous germline mutation in cyclin-dependent kinase (CDK) gene *CDKN2A* and, to a lesser extent, CDK4, *BRCA1*-associated protein 1, *MITF*, and telomerase reverse transcriptase genes.^10^ Often these individuals also display numerous melanocytic nevi. As our patient harbored a germline mutation in *ARID1B*, a gene somatically mutated in melanoma, we propose that *ARID1B* mutations might predispose to and drive the development of melanocytic nevi. Further studies are required to determine the prevalence of vitiligo/melanocytic nevus phenotype in CSS and the risk for melanoma in these individuals.

We report a patient with a genetically confirmed diagnosis of CSS and a striking phenotype affecting melanocytes, namely vitiligo and numerous melanocytic nevi. CSS requires a multidisciplinary and supportive approach. There are limited treatment options for vitiligo, with the objective to slow disease progression and promote repigmentation. For this patient, sunscreen and topical glucocorticoids were recommended. His laboratory studies were negative for thyroid disease, pernicious anemia, Addison disease, and multiple endocrinopathy syndrome. His family opted to pursue molecular analysis of his nevi to find if other predictors of malignancy are present. Those studies are pending as of the publication date of this report.

Our findings are relevant for pediatric patients presenting with vitiligo and suggest a higher index of suspicion for the risk of melanoma when melanocytic nevi are present. Last, the link between *ARID1B* mutation, vitiligo, and melanocytic nevi might facilitate identification of therapeutic targets for disorders with aberrant function or proliferation of melanocytes.
